# Pangaea Oncology, Dexeus University Hospital: bridging preclinical and clinical research

**DOI:** 10.1186/s12943-023-01927-3

**Published:** 2024-01-25

**Authors:** María González-Cao, Carlos Pedraz, Miguel Ángel Molina-Vila, Rafael Rosell

**Affiliations:** 1 IOR, Dexeus University Hospital, Barcelona, Spain; 2 Pangaea Oncology, Dexeus University Hospital, Barcelona, Spain

**Fig. 1:**
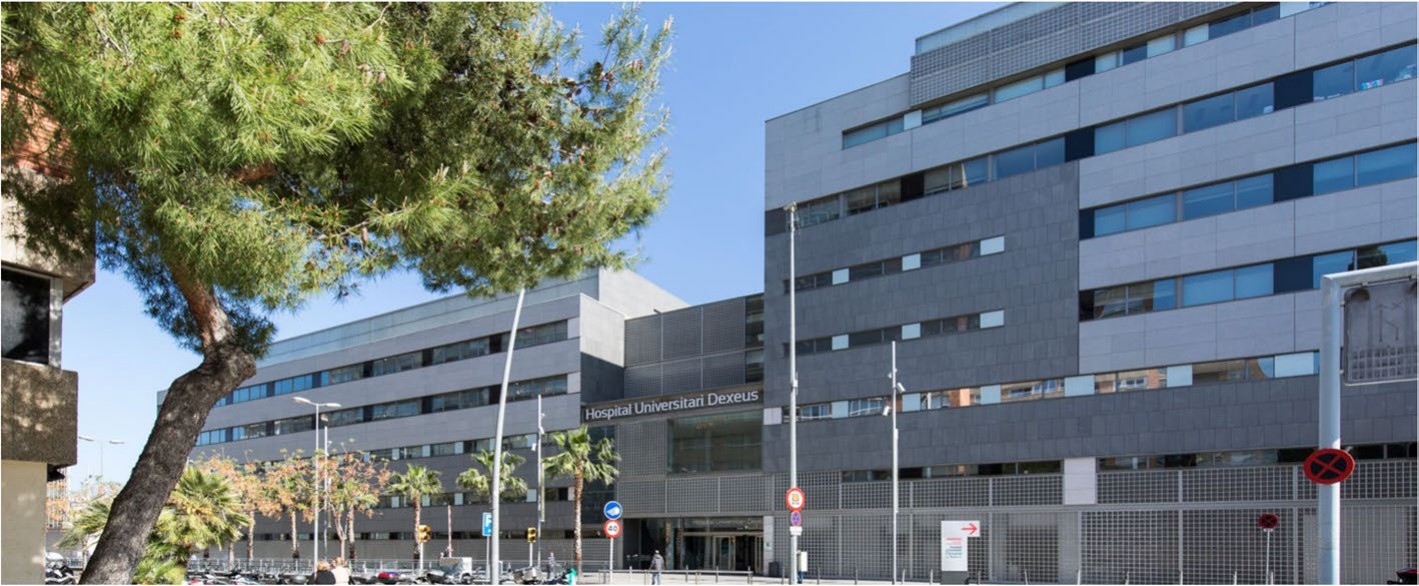
Exterior view of Hospital Universitari Dexeus, the location housing the cutting-edge laboratories of Pangaea Oncology

Pangaea Oncology has a comprehensive clinical trial portfolio: including investigator initiated trials (IITs) which emphasize Pangaea’s novel scientific development, cooperative academic studies, and industry sponsored trials. Clinical trials are the mainstay for bringing out the most promising treatment strategies and drugs to our patients, including patients that do not have private insurance or have low household incomes. Many patients are included in innovative clinical trials across Pangaea Oncology centres. As mentioned, Pangaea Oncology has a mission to improve the standard of care for oncology patients in the Barcelona area, increasing personalized enrolment in high impact trials, especially for uncommon and deadly cancers. Pangaea Oncology has increased its work in clinical trials every year and is one of the first recruiter centers worldwide in several pivotal studies that have been reflected in more than 100 publications in high impact journals that have led to novel drugs approvals in oncology, changing the standard of care. Since 2008, approximately 225 clinical trials have been opened. Over the last five years, 40 trials have commenced annually, consistently enrolling over 100 patients each year. Currently there are 110 open trials, including phase 1 (21%), phase 2 (38% ), phase 3 (30% ) and phase 4 trials (11%) Fig 1.

At Pangaea Oncology, we try to reduce the difficulties for having drug access that the current financial restrictions of the Health Public System in Spain have produced. In the majority of our IITs, patient inclusion criteria are flexible, based solely on well-defined scientific rationale. Moreover, we have developed studies for patients that are usually excluded from clinical trials, such as patients with viral infections. The DURVAST trial was a phase 1b-2 study designed for testing the activity and safety of the anti PD-L1 antibody, durvalumab, in solid tumors in individuals with HIV infection. Results of this study were presented as an oral communication in ASCO 2019 in parallel with the results of a similar study from the NCI. Results of both trials demonstrated the safety of treating with anti PD-1/PD-L1 antibodies patients infected by HIV, that had been usually excluded from cancer trials.

There are several examples of IITs which represent Pangaea cancer clinical trials based on preclinical discoveries at our lab. IITs focused on EGFR-mutant lung cancer have been one of the main pillars of Pangaea Oncology. EGFR mutations in lung cancer account for 20% of lung cancer tumors, mainly in women and non-smokers. Dr Rafael Rosell, head of the Medical Oncology Service, was one of the main investigators involved in the discovery of this disease in 2004. His research led to the establishment of a new standard of care for EGFR-mutant lung cancer patients based on the EURTAC trial, which demonstrated the superiority of the EGFR tyrosine kinase inhibitor, erlotinib, over chemotherapy. Following this success, our laboratory’s focus shifted toward understanding the molecular alterations involved in the development of drug resistance, since in most patients, these drug inhibitors achieve a duration of response shorter than 24 months. Subsequently, the phase 1–2 clinical trial EPICAL was activated, supported by the results obtained in the laboratory, demonstrating that anti–EGF antibodies significantly enhance the efficacy of tyrosine kinase inhibitors and delay the emergence of resistance in EGFR-mutant preclinical models. This trial tested the safety and activity of the combination of the second-generation EGFR inhibitor, afatinib, with an anti-EGF vaccination in EGFR-mutant lung cancer patients. We found that the combination produced immune response against EGF, and the immunized patient’s sera inhibited the EGFR pathway in vitro. Currently, a second IIT focused on EGFR non-small cell lung cancer is active: the TOTEM trial (NCT04772235), a phase 1–2 trial testing the combination of repotrectinib with osimertinib in EGFR-mutant lung cancer. The study is based on our finding that repotrectinib, a TRK/ROS1/ALK inhibitor, inhibits Src/FAC/Janus kinase 2 (JAK2), STAT3 and YAP1 signaling, abrogating tumor growth in EGFR-mutant tumors. The TOTEM trial evaluates the safety, preliminary efficacy, and pharmacokinetic and pharmacodynamic profile of repotrectinib in combination with osimertinib. Data from a phase 1 study has been presented and enrollment into the phase 2 is ongoing.

Our group has initiated another IIT in collaboration with the Immunology Unit of Hospital Clinic of Barcelona and Pompeu Fabra University: the Venezolung trial (NCT04487756), a phase 1b-2 trial evaluating the impact of an autologous dendritic cell vaccine in combination with the anti-PD-L1 antibody, atezolizumab, among patients with extensive small cell lung cancer. Small cell lung cancer is the most aggressive type of lung cancer, often associated with tobacco smoking. While its incidence has been on the decline, the persistently high mortality rate remains a challenge. Therapeutic advances in the last 40 years for these patients have been negligible. Despite recent approvals of anti-PD-1/PD-L1 antibodies for use in combination with chemotherapy, long term survivors are still uncommon. The Venezolung trial is nearing the completion of patient enrollment, with anticipated initial results expected in the last quarter of 2024. Among its exploratory endpoints, the study will analyze the evolution of cytokines, circulating lymphocyte subpopulations, and notably, alterations in dendritic cell subsets throughout treatment, as well as predictive factors of response in pretreatment tumor tissue samples. Other clinical trials that will test immune cell-based therapies as tumor infiltrating lymphocytes (TIL) will be activated in the first quarter of 2024, in addition to several clinical trials that will test the use of other advanced cancer immunotherapies, such as bispecific antibodies and TCR based biologics.

The Pangaea Oncology lab not only incorporates all the capabilities of standard pathology and tissue culture labs, but also performs comprehensive testing of > 1,000 samples per year from cancer patients. To this end, the laboratory employs state-of-the-art methodologies, including next generation sequencing (NGS), multiplex RNA-based analysis by nCounter and primary culture from fresh tumor samples. The Pangaea Laboratory was the first Spanish laboratory to receive an ISO 15,189 accreditation (European equivalent to CLIA) for genotyping of cancer samples, back in 2009; an accreditation that has been expanded over the years to cover multiplex testing by NGS and nCounter in tissue and liquid biopsies. In particular, the Pangaea Laboratory has pioneered the incorporation of liquid biopsy testing into its routine clinical practice. Currently, a multicenter study on liquid biopsy from the Spanish Melanoma Cooperative Group is being coordinated from Pangaea Laboratory, testing the concordance of different techniques for liquid biopsy analysis through four different labs in Spain. Other studies from our lab have demonstrated, for example, the prognostic role of TP53 mutations or BID overexpression as a biomarker of response to inhibitors of the spindle assembly checkpoint.

Pangaea Oncology collaborates with several leading institutions and scientific organizations, not only in Spain and Europe, but also in Latin America, India, and China. Since the inception of our group, we have welcomed foreign scientists and doctors for training periods within our laboratory and clinical teams. In total, 28 scientists, including medical oncologists, surgical oncologists, biologists, and biotechnologists, have joined us from various countries across Europe, Asia, and America. As a result of their clinical and translational research activities, Pangaea Oncology doctors and investigators have published more than 50 articles in high-impact journals, such as The Lancet, New England Journal of Medicine, and Molecular Cancer.

Our team has historically maintained cooperative interactions with patient advocates across all stages of research. Extensive collaboration with patient groups, such as the EGFR Positive Lung Cancer Resisters Advocacy Group, and The Exon 20 Group, has developed in a spontaneous way and has been reinforced throughout the years. In 2023, a special workshop focused on the ethical aspects of drug access for cancer patients was organized in Barcelona by our team. To facilitate inclusion and patient diversity, our staff represents the diverse makeup of the current Spanish population, including various races, ethnicities, ages, sexual orientations, and cultural backgrounds. The effective translation of preclinical research into patient care within our group underscores the authenticity of our organization as a private company focused on the convergence of scientific excellence and humanity in patient care.

